# Safety of meloxicam oral provocation challenge in patients with NSAID reaction

**DOI:** 10.1186/1939-4551-8-S1-A265

**Published:** 2015-04-08

**Authors:** Cristina Isabel Herrera Morales, Ricardo Cardona Villa, Catalina María Gómez Henao, Luisa María Holguín Gómez, Ruth Helena Ramirez Giraldo, Carlos Fernando Chinchilla Mejia, Libia Susana Diez Zuluaga

**Affiliations:** 1 Universidad De Antioquia, Colombia; 2 I.P.S Universitaria-Clinica León XIII, Colombia

## Background

Nonsteroidal anti-inflammatory drugs (NSAIDs) are one of the most frequently used drug groups worldwide, for their significant properties analgesic and anti-inflammatory. Induce a variety of adverse reactions, both type A (related to the effects of the drug) and type B (related to individual response). Although the mechanisms underlying the adverse effects remain unclear, inhibition of cyclooxygenase (COX) seems to be the most accepted explanation. For their significant analgesic, antipyretic and anti-inflammatory, constitute an essential medicine for the treatment of several diseases, making it imperative to find a safe alternative in patients who report a hypersensitivity reaction to AINE.

## Objective

To evaluate the safety of meloxicam as analgesic and anti-inflammatory alternative for patients who have experienced a hypersensitivity reaction to a NSAID.

## Methods

We retrospectively evaluated patients who had reported an adverse reaction to several drugs of the NSAID group and had been subjected to a challenge with Meloxicam, between 2010-2013, in our allergy service in Medellin, Colombia.

## Results

A total of fifty-seven patients reported hypersensitivity reactions to two or more NSAIDs, nineteen patients reported an anaphylactic reactions. All were challenged with meloxicam, thirty-six patients with incremental doses of 1.5, 3, 4.5 and 6 mg for a total dose of 15 mg, seventeen patients with a total dose of 7.5 mg, one patient to 10mg and one patient 5 mg for their age. Fifty-five patients had a negative test challenge, two patients (3.5%) with a positive challenge, both with skin symptoms (hives and angioedema).

## Conclusion

Meloxicam is an anti-inflammatory analgesic and safe alternative for patients who have experienced a hypersensitivity reaction to 2 or more NSAIDs.
